# An Indirect Method for Determining the Local Heat Transfer Coefficient of Gas Flows in Pipelines

**DOI:** 10.3390/s22176395

**Published:** 2022-08-25

**Authors:** Leonid Plotnikov, Iurii Plotnikov, Leonid Osipov, Vladimir Slednev, Vladislav Shurupov

**Affiliations:** 1 Department of Turbines and Engines, Ural Federal University Named after the First President of Russia B.N. Yeltsin, Str. Mira, 19, 620002 Yekaterinburg, Russia; 2 Department of Electric Drives and Industrial Installations Automation, Ural Federal University Named after the First President of Russia B.N. Yeltsin, Str. Mira, 19, 620002 Yekaterinburg, Russia

**Keywords:** heat transfer coefficient, gas flows, pipelines, thread sensor, constant-temperature hot-wire anemometer, engine intake system

## Abstract

An indirect method and procedure for determining the local heat transfer coefficient in experimental studies on the intensity of heat transfer at a gas–surface interface is described. The article provides an overview of modern approaches and technical devices for determining the heat flux or friction stresses on surfaces in the study of thermophysical processes. The proposed method uses a constant-temperature hot-wire anemometer and a sensor with a thread sensitive element fixed on the surface of a fluoroplastic substrate. A substrate with the sensor’s sensitive element was mounted flush with the wall of the investigated pipeline. This method is based on the Kutateladze–Leontiev approach (the laws of friction and heat transfer) and the hydrodynamic analogy of heat transfer (the Reynolds analogy): this is an assumption about the unity of momentum and heat transfer in a turbulent flow, which establishes a quantitative relationship between friction stresses on the heat exchange surface and heat transfer through this surface. The article presents a method for determining the speed of the developed measuring system. An example of a successful application of the proposed method in relation to the study of thermomechanical processes in the gas exchange systems of reciprocating internal combustion engines is described.

## 1. Introduction

Determining the intensity of heat transfer at the interface of two media experimentally is a widespread problem in science and technology. The main solution consists of determining the heat flux density *q_c_* = αΔ*T* and then calculating the heat transfer coefficient α. Today, there are reliable methods for measuring heat flow by creating a temperature difference between the medium (gas, liquid) and the surface (channels, pipelines) [1,2]. A large number of sensors (technical devices) and special techniques for conducting thermophysical experiments have been developed to implement these methods practically. One of the most common types of sensors are film sensors [3,4,5,6]. These sensors have a conductive film that is heated to a specific temperature to create a temperature difference between the flow and the surface. Thermocouples under the film record the temperature change over time. Yan Y.-C. et al. [3] developed a new film sensor based on a two-layer substrate structure. This made it possible to increase the sensitivity and accuracy of the measurement. For example, the accuracy of determining the heat transfer intensity increased by almost 8%. Etrati A. et al. [4,5] proposed a new thermal sensor with protective heating to improve the accuracy of measuring shear friction stresses in a turbulent flow. The relative uncertainty of the thermal experiment improved by almost 5%. Liu X. et al. [6] improved the design of traditional film sensors in order to enhance the accuracy of measuring shear friction stresses (with subsequent determination of the heat flux) during gas flow in a pipe. Determining surface heat flux with depth measurements of temperature via various thermocouples and other special devices is also widespread [7,8]. Along with a detailed study of the traditional methods for determining the intensity of heat transfer in various cases, experts also proposed original approaches for solving this thermophysical problem. For example, Hurley P. et al. [9] used fiber-optic sensors rather than thermocouples to measure temperature. The authors show decent results in measuring the temperature change in fuel rod simulators during quenching while using fiber-optic sensors. The accuracy of the new approach for determining the temperature is almost 15% higher compared to traditional methods. Wang Y. et al. [10] developed two original methods for calculating heat flux with a slug calorimeter in the field of a plasma jet: these methods increase the accuracy of predicting thermophysical processes. Alanazi M.A. and Diller T.E. [11] proposed an original, non-invasive sensor for heat energy consumption based on a combination of measurements of heat flow and temperature during gas flow in pipes. Liu Y. et al. [12] proposed a method for rapid temperature measurement using a thread sensor. This type of sensor has a higher response speed compared to film heat flow sensors. As such, thread sensors are often used to record rapidly changing (non-stationary) thermal processes. Original techniques for determining the intensity of heat transfer at the interface between two media can also be found in [13,14]. The authors propose various devices to create a temperature difference between the surface and the gas and for determining heat flux in various applications with a high degree of accuracy and speed.

Important developments in making sensors for determining heat flux and/or friction stresses are improving sensor indicators (response time, speed range, accuracy, etc.) and allowing for the change in flow temperature during experiments to be corrected. In particular, Hubble D.O. and Diller T.E. [15] developed a hybrid method for determining heat flux that improved the response time, accuracy, and versatility of the sensor. The accuracy of the proposed method is higher by 5–10% in comparison with classical approaches in thermal experiments. Experts have paid a lot of attention to methods for correcting changes in the temperature of a gas/liquid flow during experiments [16,17,18,19]. For temperature correction, various authors proposed using calibration functions to take into account gas/liquid temperatures [16], introducing correction factors [17], and employing mathematical correction methods [18,19]. The proposed correction methods significantly increase the accuracy of determining heat flux in experiments, as they consider the nonstationarity of these processes.

It should be noted that specialists have developed techniques and devices for determining the intensity of heat transfer by taking into account developments in particular scientific and technical fields. For example, one can single out studies on methods for determining heat flux at ultrahigh temperatures (more than 2000 °C) [20,21]. This can be useful in the study of hypersonic flows. Determining heat flow in spacecraft [22], the cylinders of reciprocating internal combustion engines [23,24] and complex channels [25] have also been studied separately. Several studies are related to the study of heat transfer in microchannels through numerical modeling and experimental techniques [26,27,28,29,30]. In most cases, these sensors have a high response rate, taking into account the physical characteristics of the investigated processes, and special designs applicable only to specific cases.

In most heat flux measurements, a conductive film (in particular, foil) is used to heat the surface to a certain temperature before measurements are made. In parallel with this, surface temperature is measured using thermocouples located directly under the film. Specialists focused on calibrating sensors and correcting changes in the medium’s temperature to obtain more accurate measurement results. These methods for determining heat transfer coefficients have disadvantages: (1) the large sizes of measuring devices (sensitive elements) limit the applicability of this method in many areas; (2) the influence of a large number of factors when determining the heat transfer coefficient (reliable thermal contact between the thermocouple and the surface, heat loss through the elements of the working section, thermal insulation of the non-working surface, etc.), which complicates the use of this method outside the laboratory; and (3) labor-intensive calculations of the quantitative values of the heat transfer coefficient for gas-dynamic systems with complex configurations.

This work proposes an indirect method for determining the heat transfer coefficient using a constant-temperature hot-wire anemometer and a thread sensor. This method allows for comparisons of the heat transfer rate in gas-dynamic systems with different configurations and under different boundary conditions.

## 2. Sensor Calibration

**Figure 1:**
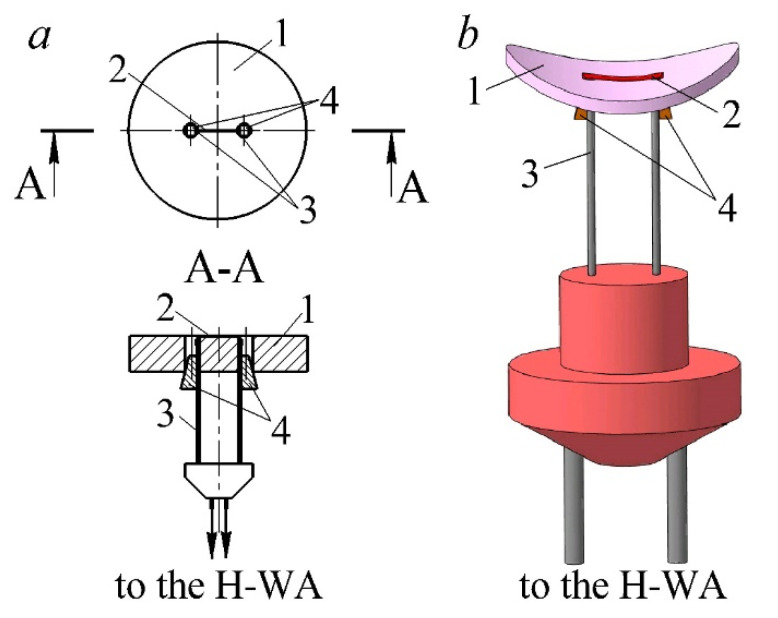
Scheme (**a**) and three-dimensional model (**b**) of the hot-wire anemometer sensor: 1—fluoroplastic substrate (thermal conductivity—0.07 W/(m·K)); 2—sensitive element of the sensor (a nichrome thread with a diameter of 5 μm and a length of 3–5 mm); 3—conductive rods; 4—wedges (wood).

The measuring system consists of two main elements—a hot-wire anemometer and a special sensor. A constant-temperature hot-wire anemometer [31] was used. The hot-wire anemometer contains a resistive temperature-sensitive element (hot-wire anemometer sensor) connected to a bridge circuit (a Wheatstone bridge) and a feedback amplifier connected to the measuring bridge. The output current of the feedback amplifier passes through the hot-wire anemometer sensor, heating it to a temperature *T*_S_ exceeding the flow temperature *T_f_*. The temperature *T*_S_ was kept constant during measurements with a feedback amplifier. The current flowing through the hot-wire anemometer sensor depends on the speed of the gas flow cooling it at given environmental parameters. The hot-wire anemometer sensor has an original design (Figure 1).

The sensor’s basic element is a fluoroplastic substrate with a thermal conductivity coefficient of 0.07 W/(m∙K). A nichrome thread with a diameter of 5 μm and a length of 4–5 mm was passed over the surface of the substrate with a slight tension. This thread is the sensitive element of the hot-wire anemometer sensor. The dimensions of the sensing element determine the speed and accuracy of the measuring system. On the one hand, the minimum thread sizes improve the performance of the measuring system. On the other hand, the accuracy of determining the local heat transfer coefficient increases if films are used instead of a thin thread. Therefore, the dimensions of the sensitive element of the sensor are determined by the scope and objectives of the study. The thread was welded to the conductive rods by annealing. Wedge-shaped clamps were used to fix the substrate and the rods, as well as to maintain tension during the experiment. The resistance of the sensing element in a cold state was 1.8–2.3 Ohms. The sensor characteristics remained stable for at least 2–3 months. This method makes it possible to give the sensor readings long-term stability during measurements [32].

The hot-wire anemometer’s thread sensor measures local friction stresses on the channel surface τ*_c_*. On the basis of τ*_c_*, it is possible to determine heat flux density and, subsequently, the local heat transfer coefficient, if the physical properties of the liquid and other flow parameters (velocity, temperature) are known. In this method, determining the local coefficient at the wall–gas flow boundary is based on the effect of the hydrodynamic analogy of heat transfer (the Reynolds analogy). This implies the unity of the impulse and heat transfer in a turbulent flow and establishes an unambiguous relationship between heat transfer and hydraulic resistance. In other words, friction on the heat exchange surface and heat transfer through this surface are interrelated [33]:

(1) $$q_{c}=\tau_{c}\frac{\lambda }{\mu }\cdot \frac{T_{f}-T_{c}}{w},$$

where *q_c_* is the heat flux density; τ*_c_* is friction stress on the surface; λ is the thermal conductivity coefficient of the gas; μ is the dynamic viscosity coefficient of the gas; *T_f_* − *T_c_* is the temperature difference between the gas and the wall; and *w* is the average gas flow velocity away from the wall.

The use of expression (1) is correct for laminar and turbulent fluid flows with a Prandl number Pr ≈ 1, as well as for cases of gas flow with physical properties independent of temperature, without internal heat sources and in the absence of mass forces.

In the proposed method, determining the local heat transfer coefficient can be done through indirect calibration using known empirical dependencies, i.e., without direct calculation of the heat flux taken from the sensor thread. This method is based on the basic index of local heat transfer for stationary heat transfer in a long straight pipe (*l*/*d* ≥ 50) with a circular cross-section. Thus, the calibration consisted of correlating the calculated heat transfer coefficient α (W/(m^2^·K)) for a long straight pipe and the value of the signal from the hot-wire anemometer sensor *U* (V). The heat transfer coefficient’s base level (Nusselt number) was calculated according to the known dependence for a turbulent flow [34]:

(2) Nu(x)d=0.022Rex0.8Prx0.43εl,

where Nu_(*x*)*d*_ = α*_x_*·d/λ*_x_*, in which α*_x_* is the local heat transfer coefficient (at a distance *x* from the entrance); *d* is the pipe’s inner diameter; and λ*_x_* is the thermal conductivity coefficient. The decisive factor is *T_x_*—the average temperature in the control section “*x*”. Pr*_x_* is the Prandl number; ε*_l_* is the correction factor for the channel length; Re*_x_* = *w*·*d*/ν*_x_* is the Reynolds number; *w* is the average speed; *d* is the pipe’s inner diameter; and ν*_x_* is the kinematic viscosity.

**Figure 2:**
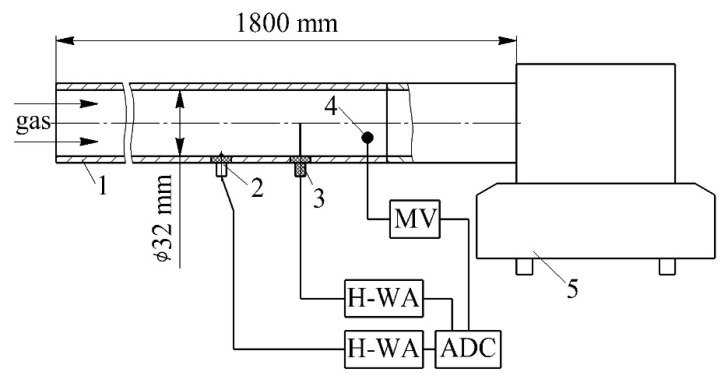
Diagram of the experimental setup for calibrating the measuring system: 1—pipeline (the length is 1.8 m and the inner diameter 32 mm); 2—hot-wire anemometer sensor for determining the local friction stress on the surface; 3—hot-wire anemometer sensor for determining the flow velocity; 4—thermocouple; 5—traction fan; MV—millivoltmeter; H-WA—electronic unit of the hot-wire anemometer; ADC—analog-to-digital converter.

To calibrate the measuring system, an experimental setup was designed, a diagram of which is shown in Figure 2.

**Figure 3:**
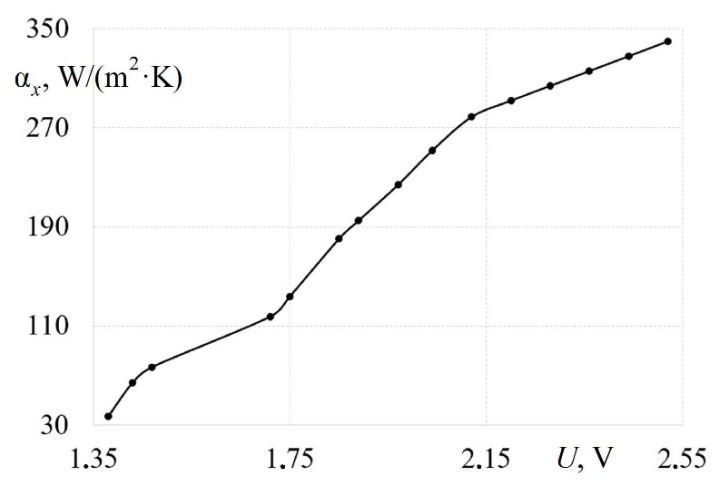
Calibration dependence for determining the local heat transfer coefficients in the pipe.

This installation included a traction fan with the ability to adjust the air flow rate by changing the engine speed. The investigated pipe had a length of 1800 mm and an inner diameter of 32 mm. Hot-wire anemometer sensors for determining the local air flow velocity and local friction stresses were installed in the pipe at a distance of 1600 mm from the entrance. The temperature was measured with a copper-constantan thermocouple placed in the channel. Data from all the sensors were fed into an analog-to-digital converter. The following values were measured when calibrating the hot-wire anemometer sensors: flow temperature, barometric pressure, local flow velocity and the voltage from the calibrated sensor. The experiments were carried out in 10–12 modes. In this case, the air flow velocity *w* varied from 0 to 120 m/s. Thus, a calibration curve was obtained in the form of a functional relationship between the voltage at the output of the hot-wire anemometer *U* and the local heat transfer coefficient α*_x_* (Figure 3). It was revealed that the calibration curve of the measuring system has an almost linear characteristic (Figure 3). However, it is necessary to take into account the different slope angle of the linear function for different ranges of air flow velocities for a more accurate determination of the heat transfer coefficient in the gas-dynamic system.

The obtained calibration dependence can be used to compare the local heat transfer in gas-dynamic systems with different configurations and at different initial boundary conditions. This approach for determining the local heat transfer coefficient is based on the Kutateladze–Leontiev method (laws of friction and heat transfer) [35,36]. This shows that it is possible to obtain the laws of friction and heat transfer for a certain standard (reference) process and then extend them to more complex cases, i.e., these laws are conservative in relation to changes in boundary conditions. A similar analogy of heat transfer (Olujic’s model) is shown in [37]. Similar conclusions can be found in [16].

## 3. Measuring System Speed

**Figure 4:**
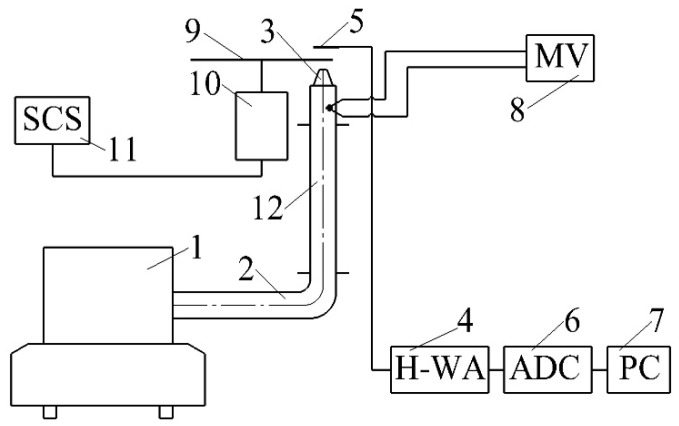
Installation diagram for evaluating the speed of the measuring system (hot-wire anemometer with a sensitive element of the sensor): 1—air blower; 2—air supply channel; 3—nozzle; 4—hot-wire anemometer; 5—sensitive element of the sensor; 6—analog-to-digital converter; 7—personal computer; 8—millivoltmeter; 9—blades; 10—electric motor; 11—speed control system; 12—section of hydrodynamic stabilization. MV—millivoltmeter; H-WA—hot-wire anemometer; ADC—analog-to-digital converter; PC—personal computer; SCS—speed control system.

Additionally, dynamic calibration was carried out to assess the speed of the measuring system (hot-wire anemometer with the proposed sensor). The evaluation of the speed of the measuring system was carried out on a laboratory installation (Figure 4). The installation consisted of an air blower with the ability to adjust the air flow by changing the engine speed. Air was supplied to the nozzle through a piping system. The gas-dynamic and heat exchange characteristics of the air were recorded by the sensitive element of the sensor located behind the nozzle. The hot-wire anemometer signal entered the analog-to-digital converter, which modified the analog signal into a binary code for further processing on a personal computer. The temperature was measured with a copper-constant thermocouple placed in the supply channel. Thermocouple readings were made using a millivoltmeter.

**Figure 5:**
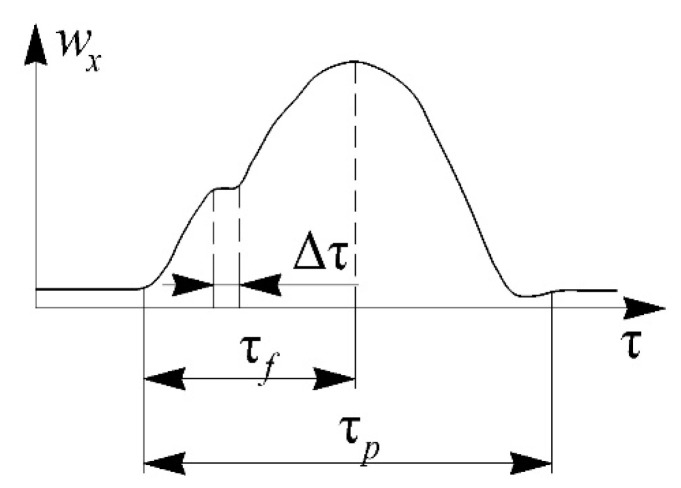
Assumed form of the curve of the air flow velocity pulsation in the gas exchange system of a reciprocating engine (simulation data): τ*_p_*—time of one pulsation; τ*_f_*—front time; Δτ—air flow fluctuation time.

Pulsations of the air flow velocity in the gas exchange system of a reciprocating engine were chosen as the basis for determining the speed of the measuring system. Previously, the expected form of the flow velocity pulsation curve was obtained by mathematical modeling in the Diesel-RK program developed at the Moscow State Technical University named after N. E. Bauman (Figure 5). It has been established that the time of one flow pulsation is approximately τ*_p_* = 60 ms at the crankshaft speed *n* = 600 rpm and τ*_p_* = 16 ms at *n* = 3000 rpm. Accordingly, the front time is τ*_f_* = 8–30 ms. The time of significant velocity fluctuation is about Δτ = 1–3 ms in the range of average flow velocity *w* from 10 to 100 m/s.

**Figure 6:**
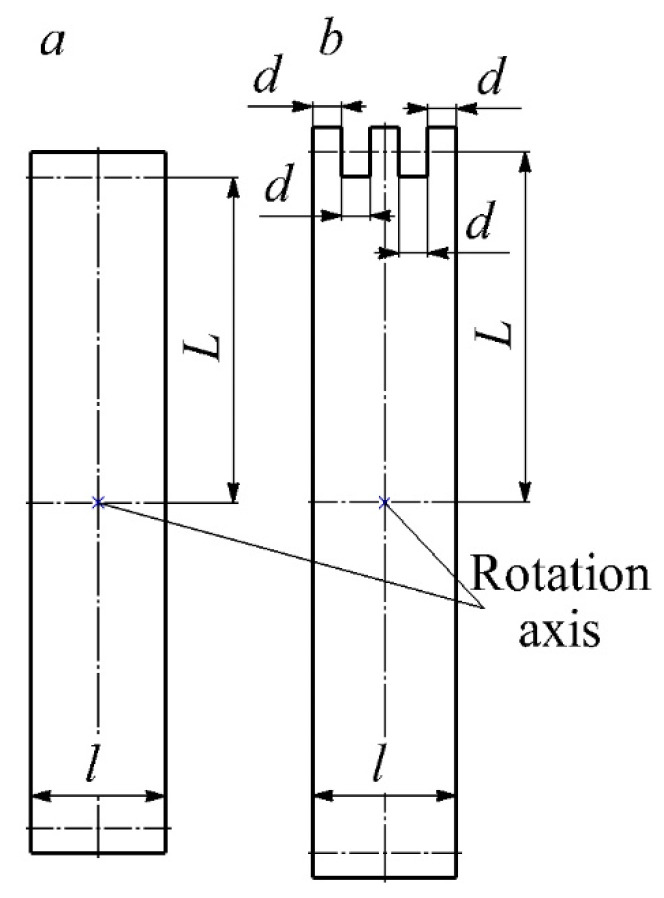
Blade configurations for dynamic calibration of the measuring system: (**a**)—one–piece blade; (**b**)—blade with slots: *l*—blade width; *L*—distance from the blade axis to the center of the hole; *d*—linear size of the slots.

The main task of dynamic calibration was to evaluate the speed of the measuring system (hot-wire anemometer with a sensitive sensor element). It is essential that the measuring system correctly capture the expected fluctuations in the air flow velocity. Dynamic calibration consisted of creating air flow pulsations by blocking the inlet channel using different blades. Two blade configurations were made (Figure 6). Slotted blades were needed to test the measuring system using the pair pulse method. The dynamic calibration of the measuring system was carried out at different blade speeds and different air flow rates.

**Figure 7:**
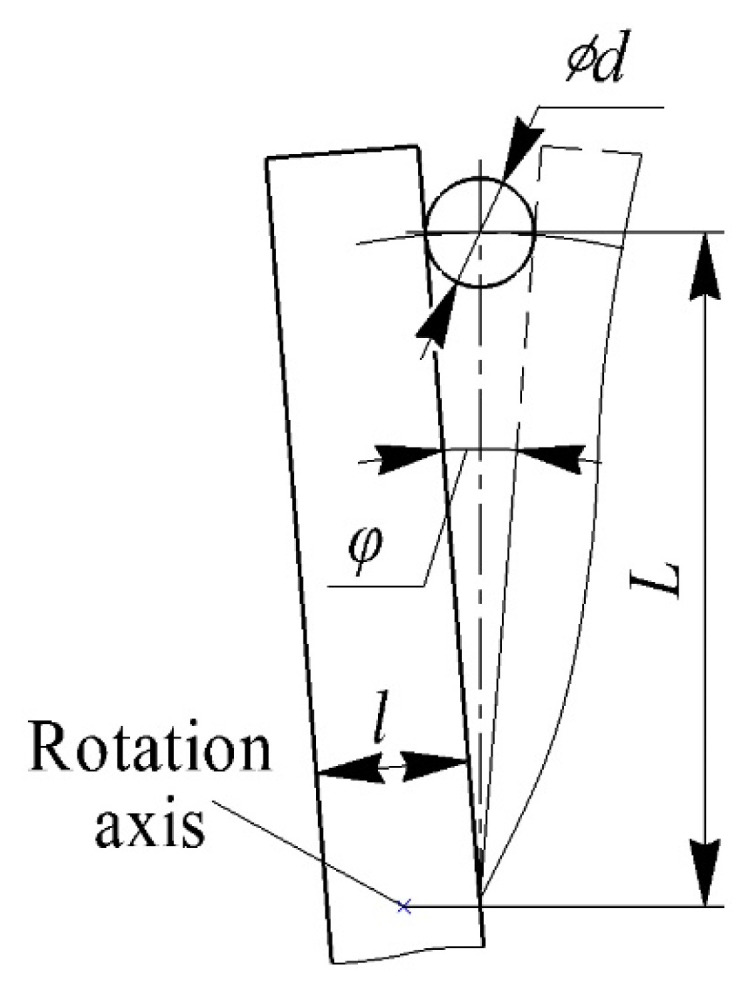
Blade overlap diagram for calculating the time during dynamic calibration of the measuring system: *l*—blade width; *L*—distance from the blade axis to the center of the hole; *d*—nozzle hole diameter; *φ*—nozzle overlap angle.

First, an analytical evaluation of the time of one pulsation τ*_p_*, the front time τ*_f_* and the steady-state time τ*_ss_* was carried out when the air flow was blocked by the blade in the laboratory installation. The blade overlap scheme is shown in Figure 7.

**Figure 8:**
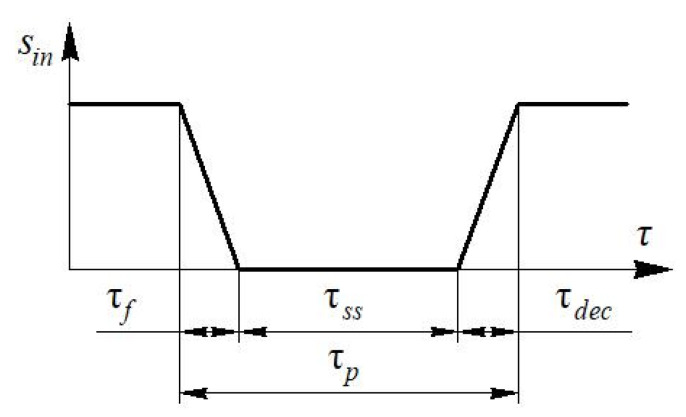
View of the input signal *S_in_* in time τ during dynamic calibration of the measuring system using a one–piece blade.

The intended form of the input signal *S_in_* from the one–piece blade is shown in Figure 8.

It is possible to obtain equations for calculating the front time and the steady-state time for the input signal based on an analytical analysis of this transient process.

Let us estimate the front time τ*_f_*—the time for which the blade completely closes the nozzle hole. The overlap angle of the nozzle φ (in radians) is equal to φ = *d*/*L* (Figure 7). Then the time of the front is equal to:

(3) $$\tau_{f}=\frac{\varphi }{2\pi f}=\frac{d}{2\pi fL},$$

where *f*—blade rotation frequency, 1/s.

Let us estimate the steady–state time τ*_ss_*—the time during which the nozzle opening is completely blocked by the blade:

(4) $$\tau_{ss}=\frac{l-2d}{2\pi fL}.$$

Obviously, the decline time τ*_dec_* is equal to the front time τ*_f_*.

**Figure 9:**
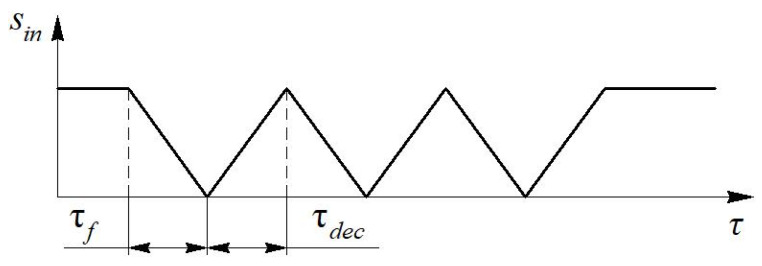
View of the input signal *S_in_* in time τ during dynamic calibration of the measuring system using a blade with slots.

The intended view of the input signal *S_in_* from the blade with slots is shown in Figure 9.

The characteristic pulsation times are τ*_f_* = τ*_dec_* = 1.7 ms, τ*_ss_* = 2 ms, τ*_p_* = 5.4 ms during rotation of a one–piece blade with parameters: *L* = 270 mm, *l* = 36 mm, *d* = 11.4 mm, *f* = 4 1/s.

**Figure 10:**
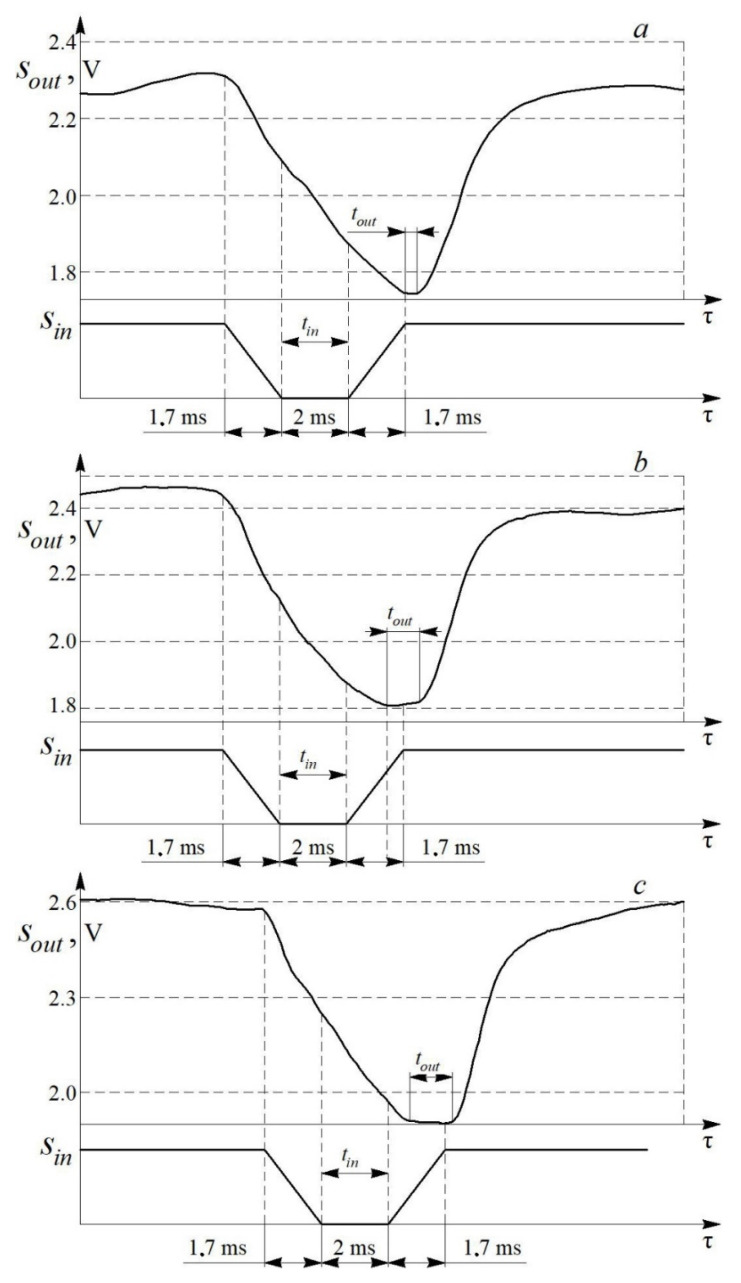
Comparison of the input *S_in_* and output *S_out_* signals of the measuring system for a one–piece blade (τ*_p_* = 5.4 ms) and at different air flow velocities *w*: (**a**)—17.8 m/s; (**b**)—28 m/s; (**c**)—40 m/s.

Comparison of the input (estimated) *S_in_* and output (obtained by the measuring system) *S_out_* signals at different air flow speeds is shown in Figure 10.

Experiments showed that the transient process does not have time to end in τ*_p_* = 5.4 ms only at a flow velocity of 17.8 m/s. This indicates that the speed of the measuring system is not enough to correctly process the input signal at low air flow rates. However, the speed is sufficient to register such flow disturbances. In this case, the measuring system registers the number and shape of pulsations, but it slightly distorts the numerical parameters. The measuring system processes the input signal with acceptable accuracy at air flow speeds above 20 m/s. The degree of correspondence between the input and output signals depends on the average flow velocity w (Figure 10). The correspondence degree should be assessed by the ratio of the characteristic linear dimensions of the output and input signals, of which the width of the signal trough (*t_in_* and *t_out_*) is the most representative. The waveform conformance factor *K_w_* is determined by the formula:

(5) $$K_{w}=\frac{t_{out}}{t_{in}},$$

where the index “*out*” corresponds to the output signal, and “*in*”—to the input signal.

**Figure 11:**
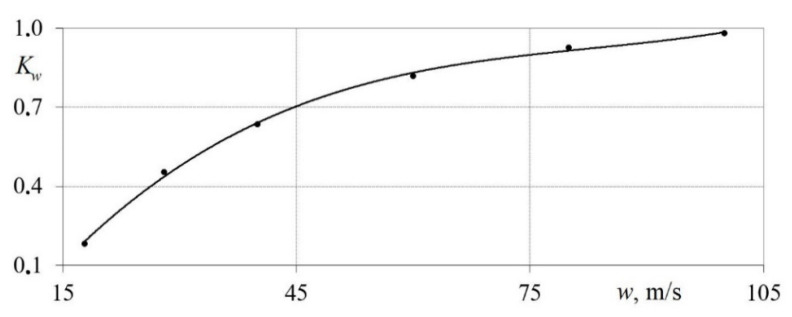
Dependence of the waveform conformance factor *K_w_* on the average air flow velocity *w*.

The dependence *K_w_* = *f* (*w*) for the measuring system is shown in Figure 11. It can be seen from the figure that the waveform conformance factor increases with a growth in the average air flow velocity.

It was found that the correspondence degree grows with an increase in air flow velocity. This indicates that the accuracy of the measuring system increases when investigating high velocity flows (*w* > 50 m/s). The lowest values of the coefficient Kw are typical for velocities in the range 5 < *w* < 20 m/s.

The time constant of the measuring system τ*_o_* can be approximately estimated by the expression [38,39]:

(6) $$\tau_{\Sigma }=\sqrt{\tau_{f}^{2}+\tau_{o}^{2}},$$

where τ_Σ_—duration of the transient process; τ*_f_*—front time; τ*_o_*—time constant of the measuring system.

**Figure 12:**
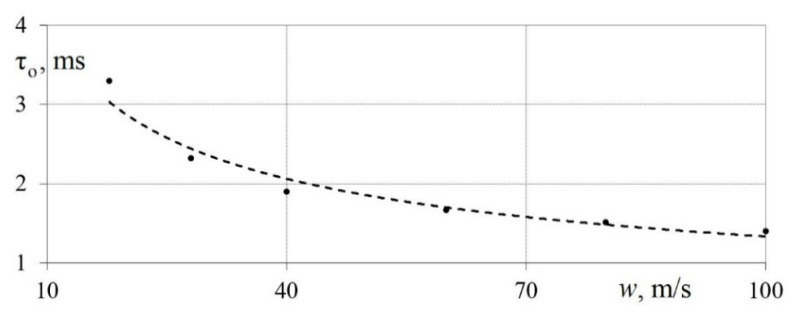
Dependence of the time constant τ*_o_* of the measuring system on the average air flow velocity *w*.

Figure 12 shows the dependence of time constant τ*_o_* of the measuring system on the average air flow velocity *w*. It has been established that the time constant decreases with an increase in the average air flow velocity. It was found that the τ*_o_* of the developed measuring system is approximately 3.0 ms at air speed *w* = 28 m/s and τ*_o_* = 2.0 ms at *w* = 40 m/s.

It can be concluded that the speed of the developed measuring system is sufficient to study gas-dynamic and heat exchange processes in heat engines in the air flow velocity range from 5 to 100 m/s. This is due to the fact that the time constant of the measuring system is about five times less than the time of the pulsation front. This satisfies the requirements of analog signal processing [40,41].

**Figure 13:**
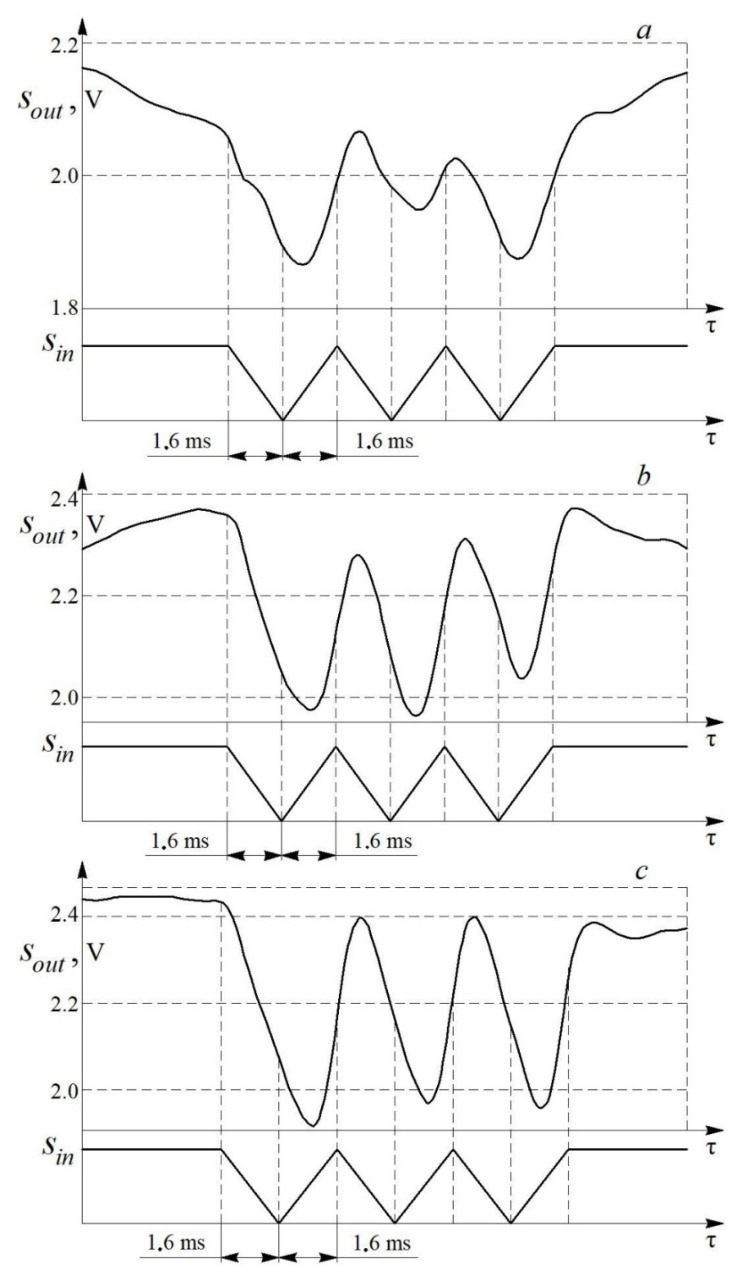
Comparison of the input *S_in_* and output *S_out_* signals of the measuring system during calibration by the method of paired pulses at different air flow velocities *w*: (**a**)—15 m/s; (**b**)—28 m/s; (**c**)—40 m/s.

Additionally, dynamic calibration by the method of paired pulses was carried out. Comparison of the input and output signals at various air speeds is shown in Figure 13. It can be seen from the figure that the measuring system correctly processes the input signal with a slight delay at speeds of 28 and 40 m/s. At the same time, the measuring system slightly distorts the input signal at a flow velocity of about 15 m/s (the system does not have enough speed to fully process the input signal).

Thus, as a result of dynamic calibration, it was found that the speed of the developed measuring system (hot-wire anemometer with a sensitive element) is quite sufficient for studying gas-dynamic and heat exchange processes in heat engines. The output signal of the measuring system correctly reflects the physical processes occurring in gas-dynamic systems in the range of air flow velocities from 5 to 100 m/s. The measuring system is able to detect small fluctuations in the flow velocity but distorts them slightly at low flow rates.

## 4. Application of the Indirect Method

**Figure 14:**
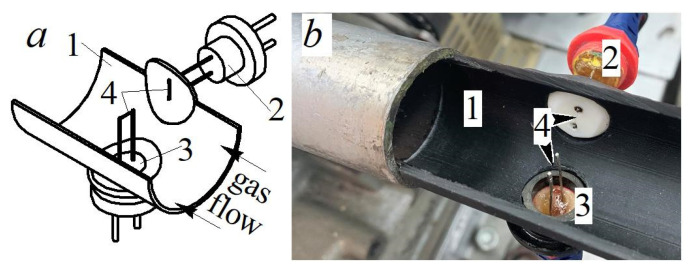
General view (**a**) and photograph (**b**) of the control section for determining local friction stress on the surface: 1—pipeline; 2—hot-wire anemometer sensor for determining local friction stress on the surface; 3—hot-wire anemometer sensor for determining the local gas flow velocity; 4—sensitive element of sensors.

**Figure 15:**
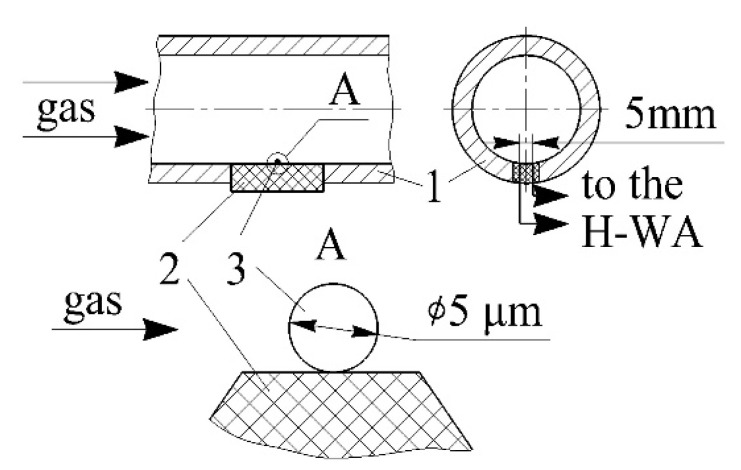
Detailed diagram of the hot-wire anemometer sensor in the pipe: 1—pipeline; 2—fluoroplastic substrate; 3—sensitive element of the sensor.

As an example, experiments comparing the values of local heat transfer coefficients in pipelines with different cross-sections as applied to the gas exchange systems of reciprocating engines were generalized [42]. An unsteady air flow in a gas-dynamic system with a complex configuration (consisting of a pipeline, a valve with obstacles and a cylinder (a cavity with a variable volume)) was investigated. A part of the pipeline (about 30% of the system’s total length) was replaced by a profiled section with a cross-section in the form of a square or an equilateral triangle. The inner diameter of the pipeline was 32 mm. The equivalent hydraulic diameter was also 32 mm for all the profiled sections. The total length of the gas-dynamic system was 0.5 m. The average air flow velocity in the system varied from 10 to 100 m/s (20,000 < Re < 200,000). The air temperature was 18–22 °C. Several control sections at different distances from the flow inlet were selected in the investigated pipeline. A hot-wire anemometer sensor was installed, the design of which is described in detail in the previous section of the article (Figure 14 and Figure 15).

**Figure 16:**
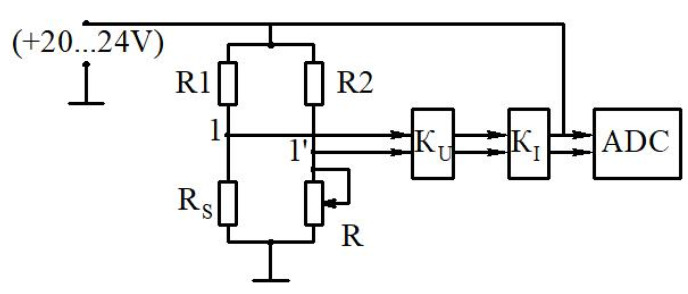
Schematic diagram of the connection of the hot-wire anemometer sensor to the power supply: ADC—analog-to-digital converter; *K_U_* and *K_I_*—voltage and current amplifiers, respectively; R, R1, R2—bridge electrical resistances, R_S_—sensor resistance.

A diagram showing the connection between the sensor and the measuring system is shown in Figure 16. The developed sensor has a resistance R_S_ and is one of the arms of the bridge for resistances R, R_1_, R_2_. The differential amplifier is connected to the measuring diagonal of this bridge. It contains the voltage amplifier *K_U_* and the current amplifier *K_I_*. The output of this amplifier supplies the diagonal of the resistance bridge. The electric current flows through the sensitive element and heats it up to 120 °C. The sensitive element’s temperature is kept constant by a servo-controlled system. The instantaneous value of the consumed electrical energy is equal to the instantaneous heat loss for heating the environment. A similar measuring system was also used in the study [43].

If the sensitive element begins to cool as a result of the flow’s heat exchange, then its resistance begins to change, which leads to a voltage drop in the diagonal of the bridge 1–1′, which is fed into the input of the amplifier. This voltage is amplified and applied to the bridge so that the amplifier current, which goes to heat the thread, increases, and compensates for its cooling. Thus, the voltage *U* (characterizing the heating of the sensor) is a measure of local friction stress on the pipeline’s surface.

A stabilized 24 V DC power supply with a current of at least 1.5 A was used to power the hot-wire anemometer. The hot-wire anemometer’s output signal was an analog signal up to 5 V, which was fed into an analog-to-digital converter.

As a result of the experiments, it was found that a change in the cross-section of the engine intake manifold reduced the intensity of local heat transfer by 5–20%, which corresponds to the results of numerical simulations in CFD systems [44] and data from other authors [45,46]. The measurement error was about 11.9%, which is quite acceptable for thermal experiments. The proposed indirect method was also used to analyze the intensity of heat transfer in the gas exchange systems of piston engines with and without supercharging [47,48].

It should be noted that the proposed indirect method can also be used to assess the intensity of heat transfer in gas and steam turbines, rotary engines, compressor technology, the nuclear industry, and other technical devices in which the working fluid is a gaseous medium.

## 5. Conclusions

Based on the research carried out, the following conclusions can be drawn:

1. An indirect method for determining the local heat transfer coefficient of gas flows in pipelines based on a hot-wire anemometer and a thread sensor is proposed. The approach’s applicability is based on the Kutateladze–Leontiev method (the laws of friction and heat transfer) and the effect of the hydrodynamic analogy of heat transfer (the Reynolds analogy).
2. The use of an indirect method, the design of a thread sensor for determining local friction stress on a surface, a method for calibrating the sensor, the main electrical parameters for connecting the sensor to the measuring system and the basic parameters of a measuring system are described.
3. It was found that the time constant of the measuring system is from 1.3 to 3.5 ms, depending on the gas flow velocity. Accordingly, the proposed method for determining the local heat transfer coefficient is applicable to the study of unsteady heat-mechanical processes in various applications.
4. The limitations of the proposed method for determining local heat transfer coefficients are discussed, and an example of using this method when building piston engines is given.
5. In the future, this research could be furthered by discussing improvements to the proposed method in terms of increasing its accuracy, expanding its applicability, and enhancing its speed and stability.

## Data Availability

Not applicable.

## Nomenclature

MV	millivoltmeter
H-WA	hot-wire anemometer
ADC	analog-to-digital converter
PC	personal computer
SCS	speed control system
CFD	Computational Fluid Dynamics
Re	Reynolds number
Nu	Nusselt number
Pr	Prandtl number
R_S_	sensor resistance, Ohms
R, R_1_, R_2_	bridge electrical resistances, Ohms
*w_x_*	local air velocity, m/s
*w*	average air flow velocity, m/s
α*_x_*	local heat transfer coefficient, W/(m^2^·K)
*p_o_*	barometric pressure, kPa
*T*	temperature, °C
*d*	pipeline diameter, mm
*l*	linear dimension, mm
*n*	crankshaft rotation frequency, rpm
*U*	electrical voltage, V
*q_c_*	heat flux density, W/m^2^
τ*_c_*	friction stress on the surface, N
λ	thermal conductivity coefficient of the gas, W/(m·K)
μ	dynamic viscosity coefficient of the gas, Pa·s
*T_f_* − *T_c_*	the temperature difference between the gas and the wall
ν*_x_*	kinematic viscosity, m^2^/s
ε*_l_*	correction factor for the channel length
τ*_p_*	time of one pulsation, s
τ*_f_*	front time, s
τ*_dec_*	decay time, s
τ*_ss_*	steady-state time, s
Δτ	time of significant pulsation, s
φ	nozzle overlap angle, deg.
*f*	blade rotation frequency, 1/s
*S_in_*	input signal, V
*S_out_*	output signal, V
*K_w_*	waveform conformance factor
*K_U_*	voltage amplifiers
*K_I_*	current amplifiers
*t_in_*	input signal trough width, s
*t_out_*	output signal trough width, s
τ_Σ_	duration of the transient process, s
τ*_o_*	time constant, s
τ	time, s

## References

[1] Gortyshov Y.F., Dresvyannikov F.N., Idiatullin N.S., Kalmykov I.I., Kovalnogov N.N., Letyagin V.G., Tonkonog V.G., Filin V.A., Khalatov A.A., Shchukin V.K. (1985). Theory and Technique of Thermophysical Experiment.

[2] Incropera F.P., DeWitt D.P. (1996). Fundamentals of Heat and Mass Transfer.

[3] Yan Y.-C., Jiang C.-Y., Chen R.-B., Ma B.-H., Deng J.-J., Zheng S.-J., Luo J. (2020). Highly sensitive flow sensor based on flexible dual-layer heating structures. Sensors.

[4] Etrati A., Bhiladvala R.B. (2014). Frequency response analysis of guard-heated hot-film wall shear stress sensors for turbulent flows. Int. J. Heat Fluid Flow.

[5] Etrati A., Assadian E., Bhiladvala R.B. (2014). Analyzing guard-heating to enable accurate hot-film wall shear stress measurements for turbulent flows. Int. J. Heat Mass Transf..

[6] Liu X., Li Z., Gao N. (2018). An improved wall shear stress measurement technique using sandwiched hot-film sensors. Theor. Appl. Mech. Lett..

[7] Wang H., Zhu T., Zhu X., Yang K., Ge Q., Wang M., Yang Q. (2021). Inverse estimation of hot-wall heat flux using nonlinear artificial neural networks. Measurement.

[8] Elkins B.S., Keyhani M., Frankel J.I. (2013). Surface heat flux prediction through physics-based calibration, Part 2: Experimental validation. J. Thermophys. Heat Transf..

[9] Hurley P., Duarte J.P. (2021). Implementation of fiber optic temperature sensors in quenching heat transfer analysis. Appl. Therm. Eng..

[10] Wang Y., Li X., Liu D., Liu Y. (2021). Analysis of Two Calculation Methods of Heat Flux Based on Slug Calorimeter. IEEE Sens. J..

[11] Alanazi M.A., Diller T.E. (2021). Noninvasive method to measure thermal energy flow rate in a pipe. J. Therm. Sci. Eng. Appl..

[12] Liu Y., Mitsutake Y., Monde M. (2020). Development of fast response heat transfer measurement technique with thin-film thermocouples. Int. J. Heat Mass Transf..

[13] Terekhov V.I., Yarygina N.I., Zhdanov R.F. (1998). Influence of External Turbulence on Heat Transfer in a Separated Flow Behind a Single Rib or a Step. Heat Transf. Res..

[14] Malyukov A.V., Mikheev N.I., Molochnikov V.M. (2016). New technique for laboratory measurements of heat transfer coefficient. Instrum. Exp. Tech..

[15] Hubble D.O., Diller T.E. (2010). A hybrid method for measuring heat flux. J. Heat Transf..

[16] Lundstrom H. (2021). Investigation of heat transfer from thin wires in air and a new method for temperature correction of hot-wire anemometers. Exp. Therm. Fluid Sci..

[17] Hultmark M., Smits A.J. (2010). Temperature corrections for constant temperature and constant current hot-wire anemometers. Meas. Sci. Technol..

[18] Manshadi M.D., Esfeh M.K. (2012). A new approach about heat transfer of hot-wire anemometer. Appl. Mech. Mater..

[19] Ardekani M.A., Farhani F. (2009). Experimental study on response of hot wire and cylindrical hot film anemometers operating under varying fluid temperatures. Flow Meas. Instrum..

[20] Pullins C.A., Diller T.E. (2012). Direct measurement of hot-wall heat flux. J. Thermophys. Heat Transf..

[21] Myrick J.A., Keyhani M., Frankel J.I. (2019). Calibration of a plug-type gauge for measurement of surface heat flux and temperature using data from in-depth thermocouples. Exp. Therm. Fluid Sci..

[22] Nenarokomov A.V., Alifanov O.M., Budnik S.A., Netelev A.V. (2016). Research and development of heat flux sensor for ablative thermal protection of spacecrafts. Int. J. Heat Mass Transf..

[23] Dejima K., Nakabeppu O. (2021). Attempt of estimating flow characteristics from wall heat fluxes measured using a three-point micro-electro-mechanical systems sensor. Int. J. Engine Res..

[24] Moussou J., Pilla G., Sotton J., Bellenoue M., Rabeau F. (2021). High-frequency wall heat flux measurement during wall impingement of a diffusion flame. Int. J. Engine Res..

[25] Liu X., Li Z., Wu C., Gao N. (2018). Toward calibration-free wall shear stress measurement using a dual hot-film sensor and Kelvin bridges. Meas. Sci. Technol..

[26] Al-Kouz W., Aissa A., Koulali A., Jamshed W., Moria H., Nisar K.S., Mourad A., Abdel-Aty A.-H., Khashan M.M., Yahia I.S. (2021). MHD darcy-forchheimer nanofluid flow and entropy optimization in an odd-shaped enclosure filled with a (MWCNT-Fe3O4/water) using galerkin finite element analysis. Sci. Rep..

[27] Jamshed W., Eid M.R., Hussain S.M., Abderrahmane A., Safdar R., Younis O., Pasha A.A. (2022). Physical specifications of MHD mixed convective of Ostwald-de Waele nanofluids in a vented-cavity with inner elliptic cylinder. Int. Commun. Heat Mass Transf..

[28] Koulali A., Abderrahmane A., Jamshed W., Hussain S.M., Nisar K.S., Abdel-Aty A.-H., Yahia I.S., Eid M.R. (2021). Comparative Study on Effects of Thermal Gradient Direction on Heat Exchange between a Pure Fluid and a Nanofluid: Employing Finite Volume Method. Coatings.

[29] Abderrahmane A., Qasem N.A.A., Younis O., Marzouki R., Mourad A., Shah N.A., Chung J.D. (2022). MHD Hybrid Nanofluid Mixed Convection Heat Transfer and Entropy Generation in a 3-D Triangular Porous Cavity with Zigzag Wall and Rotating Cylinder. Mathematics.

[30] Rasool G., Saeed A.M., Lare A.I., Abderrahmane A., Guedri K., Vaidya H., Marzouki R. (2022). Darcy-Forchheimer Flow of Water Conveying Multi-Walled Carbon Nanoparticles through a Vertical Cleveland Z-Staggered Cavity Subject to Entropy Generation. Micromachines.

[31] Orlu R., Vinuesa R. (2017). Thermal anemometry. Experimental Aerodynamics.

[32] Lundstrom H. (2015). Note: Improving long-term stability of hot-wire anemometer sensors by means of annealing. Rev. Sci. Instrum..

[33] Mukhachev G.A., Shchukin V.K. (1991). Thermodynamics and Heat Transfer.

[34] Kreith F., Black W. (1983). Basics of Heat Transfer.

[35] Tsvetkov F.F., Grigoriev B.A. (2011). Heat and Mass Transfer.

[36] Kutateladze S.S., Leontiev A.I. (1985). Heat and Mass Transfer and Friction in a Turbulent Boundary Layer.

[37] Flagiello D., Parisi A., Lancia A., Di Natale F. (2021). A Review on Gas-Liquid Mass Transfer Coefficients in Packed-Bed Columns. ChemEngineering.

[38] Stepanenko I.P. (1967). Fundamentals of the Theory of Transistors and Transistor Circuits.

[39] Mallick M., Tian X., Zhu Y., Morelande M. (2022). Angle-Only Filtering of a Maneuvering Target in 3D. Sensors.

[40] Travis J., Wells L.K. (2001). Labview for Everyone.

[41] Sindler Y., Lineykin S. (2022). Static, Dynamic, and Signal-to-Noise Analysis of a Solid-State Magnetoelectric (Me) Sensor with a Spice-Based Circuit Simulator. Sensors.

[42] Plotnikov L.V. (2022). Unsteady gas dynamics and local heat transfer of pulsating flows in profiled channels mainly to the intake system of a reciprocating engine. Int. J. Heat Mass Transf..

[43] Davletshin I.A., Mikheev N.I., Paereliy A.A., Gazizov I.M. (2019). Convective heat transfer in the channel entrance with a square leading edge under forced flow pulsations. Int. J. Heat Mass Transf..

[44] Emery A.F., Neighbors P.K., Gessner F.B. (1980). The numerical prediction of developing turbulent flow and heat transfer in a square Duct. J. Heat Transf..

[45] Rohsenow W.M., Hartnett J.P. (1973). Handbook of Heat Transfer.

[46] Terekhov V.I. (2021). Heat Transfer in Highly Turbulent Separated Flows: A Review. Energies.

[47] Plotnikov L.V., Zhilkin B.P. (2018). Influence of gas-dynamical nonstationarity on local heat transfer in the gas–air passages of piston internal-combustion engines. J. Eng. Phys. Thermophys..

[48] Plotnikov L.V. (2022). Experimental research into the methods for controlling the thermal-mechanical characteristics of pulsating gas flows in the intake system of a turbocharged engine model. Int. J. Engine Res..

